# Non-Destructive and Quantitative Evaluation of Rebar Corrosion by a Vibro-Doppler Radar Method

**DOI:** 10.3390/s21072546

**Published:** 2021-04-05

**Authors:** Takashi Miwa

**Affiliations:** Division of Electronics and Informatics, Faculty of Science and Technology, Gunma University, Tenjin-cho 1-5-1, Kiryu, Gunma 376-8515, Japan; miwa@gunma-u.ac.jp

**Keywords:** nondestructive evaluation, rebar corrosion, GPR, Doppler radar, forced vibration, displacement measurement

## Abstract

It is well known that evaluation of rebar corrosion is important for the maintenance of reinforced concrete structures, but, it is difficult to simply, quickly and quantitatively evaluate the amount of corrosion of rebars embedded in concrete by conventional non-destructive evaluation (NDE) methods such as electrical, electromagnetic and mechanical method. This paper proposes a vibro-Doppler radar (VDR) measurement method to quantitatively evaluate rebar corrosion by measuring the vibration ability of the rebar forcibly vibrated in concrete by an excitation coil. It is experimentally demonstrated in RC test pieces that the rebar vibration displacement obtained by developed VDR method is valid and is less affected by the moisture in the concrete. In addition, simultaneous monitoring of the rebar vibration displacement of the test pieces is performed through an electrolytic corrosion test and the measured vibration displacement is compared to the rebar corrosion loss evaluated. As the results, it is cleared that the rebar vibration displacement starts to increase from slightly before the occurrences of corrosion crack on the concrete surface as the corrosion loss increases. It is also shown that the rebar vibration displacement becomes 4 times higher than that in initial condition at the rebar corrosion loss of 250 mg/cm^2^. This implies that the VDR has potential to nondestructively and quantitatively evaluate rebar corrosion in concrete.

## 1. Introduction

Corrosion of rebar is a global problem that leads to deterioration of reinforced concrete (RC) structures which is one of the most versatile building materials in the world. The deterioration of concrete related to corrosion can become a safety concern, which contributes to increased maintenance costs and capital expenditures. The effect of the damage on structural behavior varies depending on the condition of the corroded concrete such as the cause and degree of corrosion. Once cracks and rust stains are observed on the concrete surface, it evidences deteriorating concrete. Missing rebar corrosion leads to serious damage by delamination. Thus, a reliable inspection method should be applied at the earliest stage possible before a lack of functionality occurs in RC structures. Moreover, quantitative evaluation of the degree of corrosion is required to aid implementation of repairs and preservation of structures for years to come, even under aggressive exposure conditions. In addition, rapidity of the inspection is also required for application to the field of health monitoring of transport infrastructures.

In order to ensure the safety of these structures, there are a variety of technologies to detect corrosion in concrete. Coring is a destructive technique in which a drilled core is extracted from an existing structure. Although they will provide the most accurate information about the health of the concrete structure, the drilled holes must be repaired afterwards. Surface potential measurements [1,2,3,4] and polarization resistance methods [5,6,7] are the most practical semi-destructive inspection methods for rebar corrosion. They can evaluate a possible existence of corrosion and the speed of corrosion, respectively. However, the rebar must be partially exposed in these methods, which also require more time for inspections. As simple non-destructive inspection (NDI) methods, electric impedance methods [8,9,10] and eddy current testing [11,12,13] are proposed for evaluation of rebar corrosion, but it is difficult to evaluate the quantitative corrosion loss of the rebar in practical situations. Thermal imaging inspection [14,15] is a non-destructive method that can quickly indicate delamination by examining changes in infrared radiation emitted from the surface of concrete. However, it is difficult to apply to bridges with asphalt overlays and in situations where there is a small thermal gradient between the bridge and the ambient temperatures.

Ground penetrating radar (GPR) [16,17] is one of the best non-destructive techniques for rebar detection in practical use because it has features of simplicity, non-contact, high spatial resolution and high contrast between the rebar and concrete. GPR has also been applied to the evaluation of rebar corrosion by using the amplitude information of the radar reflection response. It was reported that the rebar corrosion products reduce the reflection amplitude by several tens of percent once the corrosion loss is around 10% [18,19,20]. However, because moisture content and chloride content [21,22] also attenuate electromagnetic waves, compensation of their effects on the radar amplitude information is required. Therefore, despite its capability GPR only presents a qualitative assessment of the corrosion damage [23]. Thus, at present, there is no proposed reliable method that can simply and quickly, quantitatively and non-destructively evaluate rebar corrosion.

**Figure 1:**
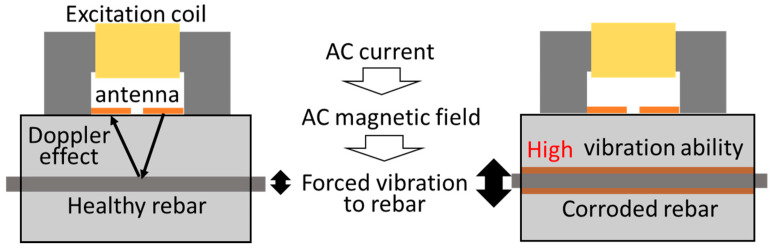
Concept of vibro-Doppler radar.

In general, a healthy rebar is strongly bonded in the concrete. However, the restraining force of the rebar weaken if rebar corrosion occurs and develops in concrete. This increases the vibration ability of the rebar in concrete. Doppler radar is a promising technique for measuring vibration displacements and is suitable for introducing it to GPR. Of course, a rebar does not vibrate by itself, so an exciting coil will allow a steel bar to display a measurable vibration displacement as shown in Figure 1. If we can nondestructively measure the vibration displacement of the rebar forcibly vibrated in concrete, the vibration displacement indirectly will become an indicator of the rebar corrosion. This method, called the vibro-Doppler radar (VDR) method, can be proposed as a novel and practicalway to resolve this problem for GPR because the vibration displacement is considered to be less affected by moisture content.

In this paper, a VDR measurement system is proposed which can non-destructively measure vibration displacements of a rebar using the principle of Doppler radar. The validity of this method is experimentally verified using RC test pieces. In addition, the relationship between the amount of corrosion loss and the rebar vibration displacement is quantitatively discussed by monitoring the rebar vibration displacement through an electrolytic corrosion test.

## 2. Principle of the Vibro-Doppler Radar Method

Vibration detection by using a Doppler radar method is a fundamental technique. Since the vibration displacement produces a phase modulation in a received sinusoidal wave, it is proportional to the time variation of the phase. If the received signal is a pulsed wave, the vibration displacement at a specific distance can also be obtained. In a general pulsed Doppler radar, the time variation of the phase can be obtained with a pulse repetition rate sufficiently faster than that of the vibration. In recent years, this has been widely applied to non-contact vital sign measurements [24,25] and structural health monitoring [26]. Because these applications focus on the motion of the vibration, FMCW systems are widely used with millimeter wave bands which have wavelengths similar to the vibration displacement [24,25]. On the other hand, when using this method for evaluating the vibration ability of a rebar, the magnitude of the vibration is essential rather than the motion. However, the vibration displacement is expected to be about 1/1000 times smaller than the wavelength used in GPR, therefore, the measurement system in VDR requires a higher dynamic range than that used in other applications. Since the forced vibration in VDR can be limited to a single frequency, only the specific Doppler frequency needs to be considered. Thus, a higher dynamic range can be expected by introducing a principle of lock-in amplifier into network analyzer measurement.

**Figure 2:**
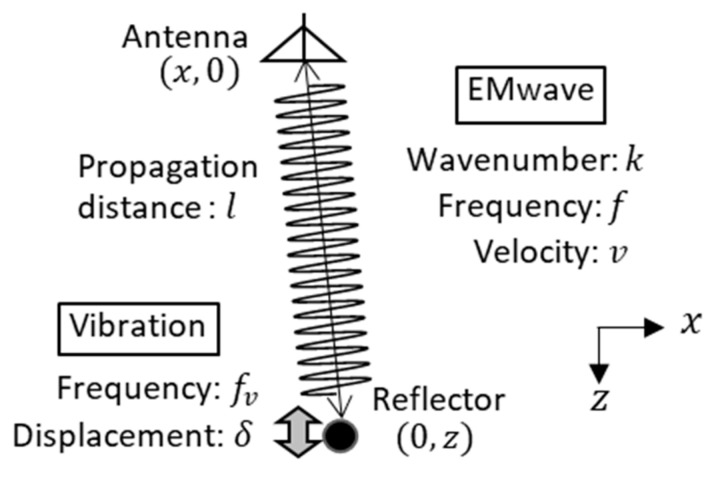
Geometry of problem.

Figure 2 shows a conceptual diagram of a displacement measurement by a vibro-Doppler radar. Assuming that a reflector vibrates with a single frequency $f_{v}$, the displacement u(t) of the reflector in the path direction is defined as:

(1) $$u\left(t\right)=\delta \cos\left(2\pi f_{v}t\right).$$

Here, δ means the vibration displacement of the vibrating reflector. In addition, assuming that l is the one-way distance from an antenna to the static reflector, the actual distance L is expressed with a function of time as:

(2) L(t)=l−u(t).

For simplicity, we consider Doppler effect in electromagnetic (EM) wave propagation at a single frequency. Assuming that the EM wave is emitted from the antenna toward the reflector and that the reflected wave from the reflector is received by the same antenna, the received complex signal $\dot{e}\left(f,t\right)$ is expressed by ignoring the antenna characteristics and the spherical diffusion term as:

(3) $$\dot{e}\left(f,t\right)=\dot{R}e^{j\left\{2\pi ft-k2L\left(t\right)\right\}}.$$

Here, $\dot{R}$ is complex reflection coefficient of the reflector and k is a wavenumber 2πf/v of the EM wave. Since the propagation path length of the reflected wave fluctuates slightly in time due to vibration, the following equation can be given as:

(4) $$\dot{e}\left(f,t\right)=\dot{R}e^{j\left(2\pi ft-2kl\right)}e^{j2k\delta \cos\left(2\pi f_{v}t\right)}.$$

Here, v means the propagation velocity of the EM wave.

Assuming that the vibration displacement is sufficiently smaller than a wavelength λ of the EM wave, kδ=2πδ/λ is much smaller than the unity. Therefore, the received signal $\dot{e}\left(f,t\right)$ can be approximated as:

(5) $$\dot{e}\left(f,t\right)≅{\dot{e}}_{0}\left(f,t\right)+{\dot{e}}_{d}\left(f,t,f_{v}\right)+{\dot{e}}_{d}\left(f,t,-f_{v}\right),$$

by performing Taylor expansion for kδ in the vicinity of kδ=0 and by neglecting the terms higher than the second order. Here, the signal ${\dot{e}}_{0}\left(f,t\right)$ is represented as:

(6) $${\dot{e}}_{0}\left(f,t\right)=\dot{R}e^{j\left(2\pi ft-2kl\right)},$$

which is equivalent to the received signal from the stationary target and is referred to as an unmodulated component.

Furthermore, a transfer function of the unmodulated component ${\dot{G}}_{0}\left(f\right)$, which is obtained by extracting only the components of frequency f from Equation (6) by orthogonal detection, is expressed as:

(7) $${\dot{G}}_{0}\left(f\right)=\dot{R}e^{-j2\pi f2l/v}.$$

In general, Equation (7) does not have spatial resolution. but we can easily obtain spatial resolution by measuring the transfer function ${\dot{G}}_{0}\left(f\right)$ in wide frequency range having the center frequency $f_{c}$ and the bandwidth $f_{w}$. If we take inverse Fourier transformation of ${\dot{G}}_{0}\left(f\right)$, the impulse response ${\dot{g}}_{0}\left(t\right)$ in the unmodulated component is given as:

(8) $${\dot{g}}_{0}\left(t\right)=\frac{1}{f_{w}}\int_{f_{c}-\frac{f_{w}}{2}}^{f_{c}+\frac{f_{w}}{2}}{\dot{G}}_{0}\left(f\right)e^{j2\pi ft}df=\dot{R}\sin c\left\{\pi f_{w}\left(t-\frac{2l}{v}\right)\right\}e^{-j2\pi f_{c}\left(t-\frac{2l}{v}\right)},$$

which corresponds to a complex radar waveform for a static target. 

On the other hand, the reflected wave from the vibrating object is modulated by the vibration frequency of ±$f_{v}$ due to the Doppler effect of the vibration. Thus, the Doppler signal ${\dot{e}}_{d}\left(f,t,\pm f_{v}\right)$ appears in Equation (5), which is given by:

(9) $${\dot{e}}_{d}\left(f,t,\pm f_{v}\right)=jk\delta \dot{R}e^{j\left\{2\pi \left(f\pm f_{v}\right)t-2kl\right\}}.$$

Generally, a network analyzer can measure frequency components at the transmission frequency with a narrow bandwidth called as an intermediate frequency (IF). If the modulating frequency is over the IF bandwidth, it is difficult to obtain the Doppler component. In order to measure only the Doppler component with a network analyzer, the transmitting frequency f should be shifted by the Doppler frequency $f_{v}$ outside the network analyzer in advance. The modulated signal having the frequency of $f-f_{v}$ is demodulated to the frequency f by reflection from the vibrating reflector. Because L(t) can be regarded as l from δ≪l, the following equation is obtained:

(10) $$\begin{array}{ll} {\dot{e}}_{d}\left(f-f_{v},t,+f_{v}\right) & =jk\delta \dot{R}e^{j2\pi f\left(t-\frac{2L\left(t\right)}{v}\right)}\\ & ≅jk\delta \dot{R}e^{j2\pi f\left(t-2l/v\right)}, \end{array}$$

which is called as a Doppler component. Thus, the transfer function of the Doppler component ${\dot{G}}_{d}\left(f\right)$ can be expressed as:

(11) $${\dot{G}}_{d}\left(f\right)≅j2\pi f{\dot{G}}_{0}\left(f\right)\delta /v.$$

Therefore, an impulse response ${\dot{g}}_{d}\left(t\right)$ in the Doppler component is given by inverse Fourier transformation of ${\dot{G}}_{d}\left(f\right)$ as:

(12) $${\dot{g}}_{d}\left(t\right)=\frac{\delta }{v}\frac{d}{dt}{\dot{g}}_{0}\left(t\right),$$

which is a complex radar waveform for the vibrating target.

Here, the absolute waveform of the unmodulated component ${\dot{g}}_{0}\left(t\right)$ has a peak at the round-trip time t=2l/v. In addition, the positive Doppler component ${\dot{g}}_{d}\left(t\right)$ is proportional to the vibration displacement δ and is expressed by the time derivative waveform of the unmodulated component. Therefore, estimated vibration displacement $\tilde{\delta }\left(l\right)$ of a reflector placed at the one-way distance l is given with the amplitude ratio as:

(13) $$\tilde{\delta }\left(l\right)=v\left|{\dot{g}}_{d}\left(2l/v\right)/\frac{d}{dt}{\dot{g}}_{0}\left(2l/v\right)\right|.$$

We note that Equation (13) is the vibration displacement obtained by waveform-based estimation and is applicable when there is only one isolated reflector within the time resolution of the radar waveform.

## 3. Measurement of Vibration Displacement by Vibro-Doppler Radar

### 3.1. Vibro-Doppler Radar System

**Figure 3:**
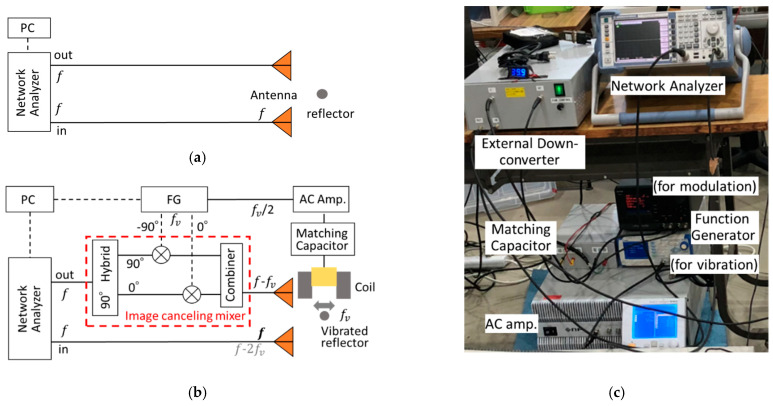
Block diagram of vibro-Doppler radar system based on network analyzer: (**a**) for non-modulation component; (**b**) for Doppler modulation component; (**c**) Photo of developed vibro-Doppler radar measurement system.

The developed vibro-Doppler radar system is based on frequency swept continuous wave radar using a network analyzer. Figure 3a shows a configuration for measuring the unmodulated component, which is equivalent to a normal step frequency continuous wave radar. Figure 3b also shows a configuration for measuring only the Doppler component, which is realized by an image cancelling mixer (ICM) as shown in the red frame. In the ICM, the input signals separated by power divider are modulated with orthogonal signals for sinusoidal vibration by the mixers (ML1-0113, Marki Microwave, Morgan Hill, CA, USA). The modulated signals are synthesized by the power combiner (ZFRSC-123, Mini-circuits, Brooklyn, NY, USA), resulting in the fact that only a sideband modulated component is obtained. The frequency shift in the ICM is only around 100 Hz for GHz band frequencies, so a part of the input signal also leaks to the output port without shifting. Since the leakage signal has the same frequency as the desired Doppler modulation component, it should be subtracted from the received signal. On the other hand, when the coil does not work, only the leakage signal can be measured with the same system configuration. Therefore, it can be cancelled by taking the difference between radar responses with and without forced vibration.

The excitation coil has a U-shaped directional laminated electrical steel sheet core (35H360A) and is wound by 500 turns of 12 mm enameled wire with heat resistance up to 240 °C. The coil has the inductance of 111 mH and the DC resistance of 1.9 Ω. A sinusoidal wave having half the vibration frequency was applied through a constant current AC amplifier to the excitation coil. A matching capacitor of 70 μF is connected in series with the coil for cancelling the reactance. When the excitation frequency to be the resonance frequency of 57 Hz, the rebar vibrates at 114 Hz because the positive and negative alternating magnetic fields generates tensile force. Figure 3c shows an overview of a developed vibro-Doppler radar measurement system.

### 3.2. Rebar Vibration Displacement in Air with Laser Displacement Sensor

**Figure 4:**
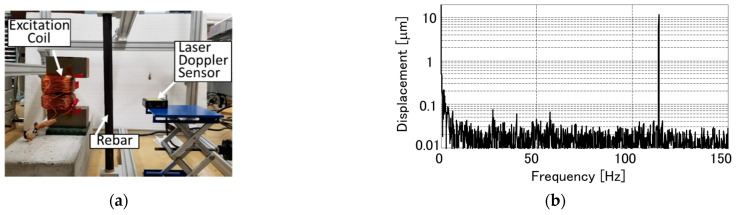
Rebar vibration characteristics in air: (**a**) Measurement setup; (**b**) Displacement spectrum obtained by a laser displacement sensor (LDS).

The validity of this system is demonstrated through experiments. At first, vibration characteristics of a forcibly vibrated rebar is discussed with a laser displacement sensor (LDS). Figure 4a shows the measurement setup. The used cylindrical rebar has a diameter of 19 mm, and both ends of the rebar were tightly fixed. The excitation coil separated by 40 mm from the rebar was excited with the applied current of 10 A and with the frequency of 57 Hz. The rebar vibration displacement was measured for 10 s by a LDS with a focal length of 85 mm from the opposite side to the coil. Figure 4b shows displacement spectrum by taking Fourier transformation of the displacement waveform. A sharp peak can clearly be observed only at the vibration frequency of 114 Hz. Table 1 shows the displacement measured in the various excitation current. As the current increases, the displacement also increases. This system achieved the vibration displacement of 11.5 μm at the excitation current of 10 A although the rebar was separated by even 40 mm from the excitation coil.

### 3.3. Rebar Vibration Displacement in Concrete with Laser Displacement Sensor

**Figure 5:**
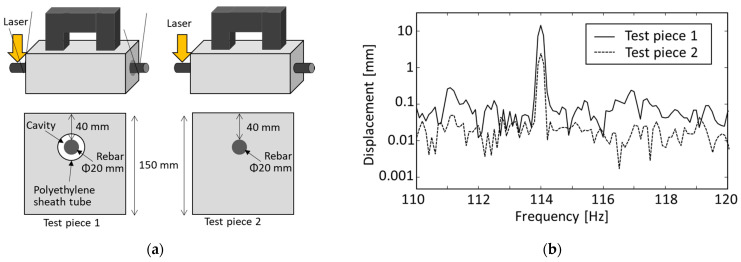
Measurement of rebar vibration displacement in concrete: (**a**) Experimental setup; (**b**) Displacement spectrum measured at the end of the rebar.

Next, a vibration displacement is evaluated in a reinforced concrete by LDS. Figure 5a shows an outline of the experiment. The used concrete test pieces are 150 mm × 150 mm × 400 mm in size. Test piece 1 has a cylindrical cavity with a diameter of 40 mm at the concrete cover of 30 mm. A rebar with the diameter of 19 mm was inserted at the depth of 40 mm and was suspended in the center of the cavity by strings at the both ends as shown in the figure. The test piece 2 is a reinforced concrete with a rebar with a diameter of 19 mm (D19) embedded at the concrete cover of 40 mm. Although the rebar of the test piece 1 vibrates without any restriction, that of the test piece 2 cannot freely vibrate. When the rebar was forcibly vibrated by the excitation coil with the same condition, the vibration displacement waveform was measured with the LDS at the end of the rebar for each test piece. Figure 5b shows measured displacement spectrum. We can observe a sharp peak at the vibration frequency of 114 Hz in each case. The vibration displacement of the test piece 1 is 13.4 μm, which is the same order as that of the previous experiment in the air. Since the LDS is mechanically isolated from the vibration system between the coil and the rebar, it is considered that the LDS can measure the vibration from rebar itself. Moreover, the sinusoidal vibration from the embedded rebar is also observed in test piece 2. In spite of the fact that the rebar is embedded in the concrete, a vibration displacement of 2.5 μm is achieved at the end of the rebar. It is expected that the vibration displacement of the embedded rebar could have increased if we could measure the vibration displacement just below the coil.

### 3.4. Measurement of Rebar Vibration Displacement with Vibro-Doppler Radar

**Figure 6:**
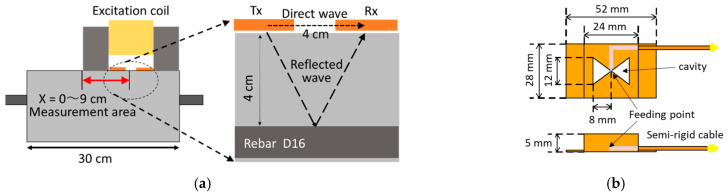
Measurement setup for VDR in concrete: (**a**) Antenna arrangement (**b**) Configuration of used antenna.

At first, the vibration measurement by vibro-Doppler radar (VDR) is discussed for a sound reinforced concrete test piece. The test piece was dried with an oven at 60 °C for 24 h before the measurement. Figure 6a shows the measurement setup for VDR measurements in concrete. The rebar, with a diameter of 16 mm, is embedded in a concrete cover of 40 mm. The excitation coil was placed on the surface of the test piece just above the rebar. Tapered slot antennas with a cavity back were used for transmission and reception. The configuration of the antenna is shown in Figure 6b. They were also fixed to the coil at the center of both legs of the coil with a feed point spacing of 50 mm. The coil and antennas were scanned above the rebar for 10 cm section and performs VDR measurement every 1 cm. A network analyzer measured the antenna transfer functions for both unmodulated and Doppler components. The measurement setup is summarized in Table 2. The complex radar waveforms were obtained by inverse Fourier transformation of them.

**Figure 7:**
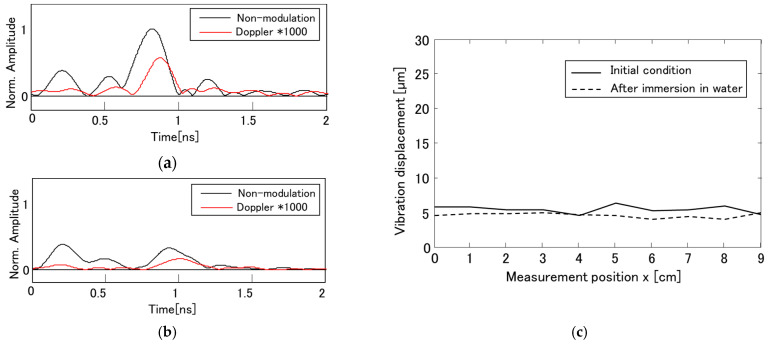
Results of Vibro-Doppler radar (VDR) measurement in concrete: (**a**) Envelope of VDR waveforms obtained for initial condition of test piece; (**b**) Envelope of VDR waveforms after immersion of test piece in water; (**c**) Vibration displacement of the rebar embedded in concrete.

Figure 7a shows the envelope of the complex radar waveforms obtained by the VDR measurement. The black and red lines correspond to the unmodulated component ${\dot{g}}_{0}\left(t\right)$ and the Doppler component ${\dot{g}}_{d}\left(t\right)$, respectively. The amplitude of the Doppler component is multiplied by 1000. In the unmodulated component, a small peak is observed at around 0.2 ns which corresponds to a wave directly propagating between the antennas as shown in Figure 6a. A distinct peak is observed at around 0.8 ns, whose arrival time corresponds to the propagation delay determined from the geometrical arrangement between the antennas and the rebar when the relative permittivity of the concrete is 7. This indicates that it is the expected reflected wave from the rebar. On the other hand, the Doppler component has only a single peak from the rebar around 0.8 ns. The direct wave in the Doppler component is much lower than that in the unmodulated component because the antennas made of non-magnetic copper is less affected by the exciting coil. Therefore, the VDR can measure the vibration from the embedded rebar having the concrete cover of 40 mm with a sufficient SNR.

Next, the effect of moisture content is discussed on a measurement of the rebar vibration displacement. The test piece was immersed in water for 5 h, resulting that the weight of the test piece increased by 1.7%. The vibration displacement of the rebar was measured immediately after wiping off the water on the concrete surface. The results are shown in Figure 7b. The arrival time of the reflected wave from the rebar delays due to increase of moisture inside the concrete. The amplitude of the reflected wave in the wet state gets smaller than that in the dry state because the electromagnetic wave significantly attenuates by moisture. However, the amplitudes of the reflected waves decreased for both the unmodulated and Doppler components, indicating that the amplitude ratios do not change much.

The rebar vibration displacements are evaluated by scanning the VDR on the concrete surface along the rebar. Figure 7c shows the results obtained from Equation (13) before and after water immersion. The vibration displacement before water immersion is about 5 μm, which has almost the same order of the vibration displacement measured with the LDS shown in Figure 5b regardless of the VDR position.

Moreover, it is found that the vibration displacement does not significantly change after water immersion. Although the moisture content significantly affects the reflection amplitude of the rebar, the rebar vibration displacement is almost same depending on the moisture content in the concrete. This is one of the great advantages of the VDR for evaluating rebar corrosion in a practical situation. 

## 4. Measurement of Rebar Vibration Displacement in Electrolytic Corrosion Test

### 4.1. Overview of the Electrolytic Corrosion Test

It is quite important to evaluate the relationship between vibration ability and rebar corrosion in concrete. Vibration displacement is considered as one of the quantitative measures of the vibration ability. It is well known that an electrolytic corrosion test can accelerate the rebar corrosion. The amount of rebar corrosion can be obtained by measuring the mass of the rebar which has been extracted from the concrete. Therefore, rebar vibration displacement is monitored while the electrolytic corrosion test in order to quantitatively evaluate the relationship between vibration displacement of the rebar and amount of the rebar corrosion.

**Figure 8:**
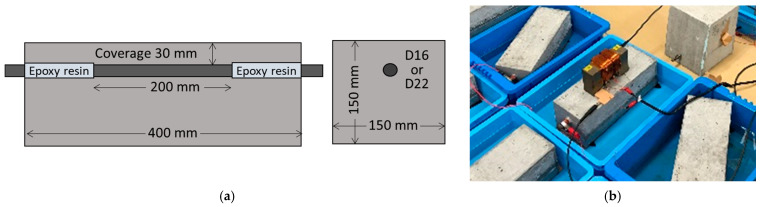
Overview of experiment of: (**a**) Fabricated RC test piece; (**b**) Electrolytic corrosion test.

Figure 8a shows overview of RC test pieces used in this experiment. Table 3 shows the concrete mixing, the fresh property of the concrete and the mechanical property of the test piece. Ordinary Portland cement was used for concrete which had a water to cement ratio of 55%. The test pieces are 400 mm wide, 150 mm high and 150 mm deep. The concrete has one embedded rebar whose both ends are sealed with epoxy resin, so the corrosion area can be 200 mm. The concrete cover of all the test pieces is 30 mm. The test pieces have two kinds of rebar diameter level of 16 mm(D16) and 22 mm(D22). The number (N) of test pieces is five in each level. All the test pieces were wet-cured for 28 days, resulting in an averaged compressive strength of 41.2 N/mm^2^.

Figure 8b shows a photo of the electrolytic corrosion test. The test pieces were placed into saline solution of 6% concentration. The solution height was kept to be 8 cm from the bottom of the test piece. A copper plate of 150 mm × 400 mm was immersed between the test piece and the case with the spacing of 5 mm.

**Figure 9:**
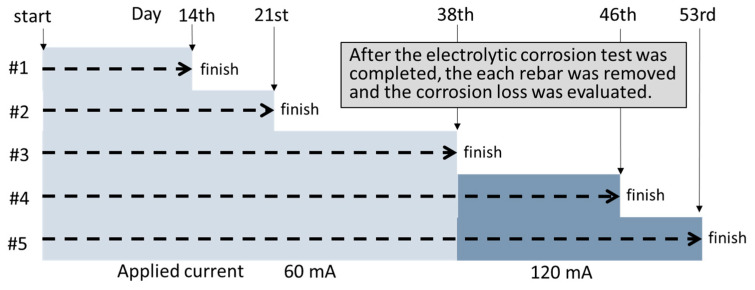
Protocol of the electrolytic corrosion test.

Figure 9 shows protocol of the electrolytic corrosion test. A DC current of 60 mA was simultaneously applied between the rebar with positive polarity and the copper plate with negative polarity for all the test pieces until the 38th day. After the 39th day, the current was increased to 120 mA to accelerate the rebar corrosion. The electrical corrosion was terminated in stages for each test piece on the 14th, 21st, 38th, 46th, and 53rd day. During the electrolytic corrosion test, VDR measurements were conducted once a day for all the RC test pieces in order to monitor the rebar vibration displacement. The VDR was placed directly on the test piece surface along the rebar as shown in Figure 8b.

**Figure 10:**
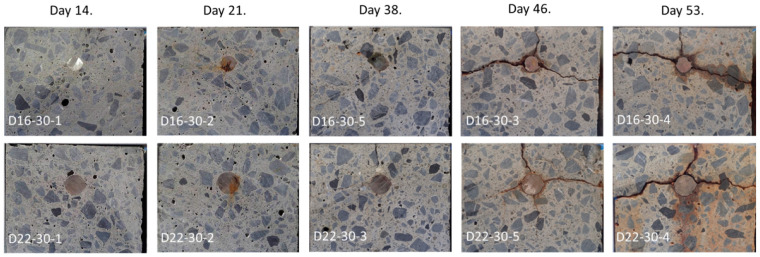
Cross-sectional photos of test pieces after terminating the electrolytic corrosion test.

Figure 10 shows cross-sectional views of the test pieces after cutting them into two parts. Although no apparent crack is observed until the 38th day, corrosion cracks appear in the lateral and upward direction starting from the 46th day. By the 53rd day, we can observe much wider cracks and much more corrosion products in the crack.

**Figure 11:**
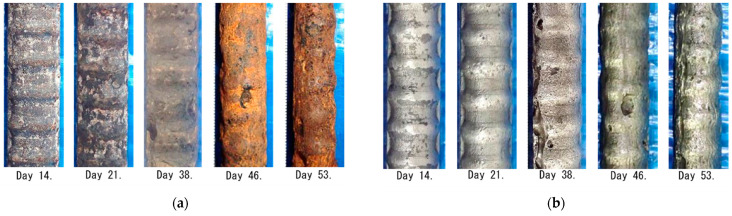
Photographs of corrosion situation of the rebar taken out from the test pieces: (**a**) before and; (**b**) after removing the rust from the rebar.

In order to obtain corrosion loss of the rebar due to the test, at first, the rebar was taken out from the test piece. And then the rust was removed from the rebar. Figure 11a,b show photos of corrosion situations of the rebar taken out the concrete before and after removing the rust, respectively. Before the 21st day, rust does not clearly adhere to the rebar. On the other hand, the rebar is covered with rust after the 38th day, resulting in reduction of knots and ribs of rebar and occurrence of pitting corrosion. In particular, it is observed that the rebar diameter decreases significantly by the 53rd day.

The corrosion loss was calculated by comparing the mass of the rebar between before and after the electrolytic corrosion test. Table 4 shows the evaluated corrosion loss. 

Although the corrosion loss was small before the 21st day, it suddenly increased as the cumulative current increases. Finally, the corrosion loss reached to 14.6% and 7.7% for the rebar diameter of 16 mm and 22 mm, respectively.

### 4.2. Curve Fitting of Corrosion Loss as a Function of Cumulative Current

**Figure 12:**
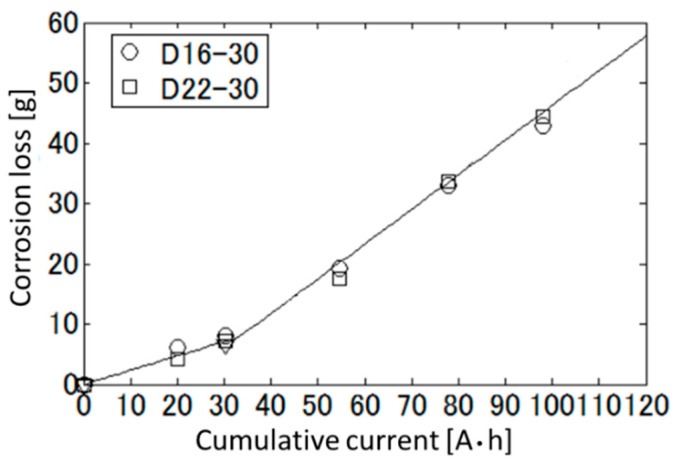
Relationship between the corrosion loss of the rebar and the cumulative current in the electrolytic corrosion test.

Figure 12 shows the relationship between the measured corrosion loss of the rebar and the integrated current obtained for each test piece. Although, in general, the amount of rebar corrosion in electrical corrosion is proportional to the cumulative current, the corrosion loss of each test piece are plotted on a bend line from the figure. Up to the cumulative current of 30 Ah, the corrosion loss is approximated to be a line passing through the origin written as:

(14) $$M_{rust}=0.237Q_{in}\;(Q_{in}<30\;\mathrm{Ah}),$$

where $M_{rust}$ and $Q_{in}$ mean the corrosion loss [g] and cumulative current [Ah]. In the cumulative current of more than 30 Ah, it is approximated to be the straight line with intercept as:

(15) $$M_{rust}=0.575Q_{in}-11.226.\;(Q_{in}\geq 30\;\mathrm{Ah}).$$

The increase of the slope of the straight line indicates an acceleration of corrosion. Since cracking to the concrete surface increases the air permeability inside the concrete, we guess that the cracking of the test pieces occurred at about 30 Ah of cumulative current. The cumulative current in this experiment is transformed into the estimated corrosion loss of the rebar by using this approximated bent line. 

### 4.3. Result of Rebar Vibration Displacement by Vibro-Doppler Radar

**Figure 13:**
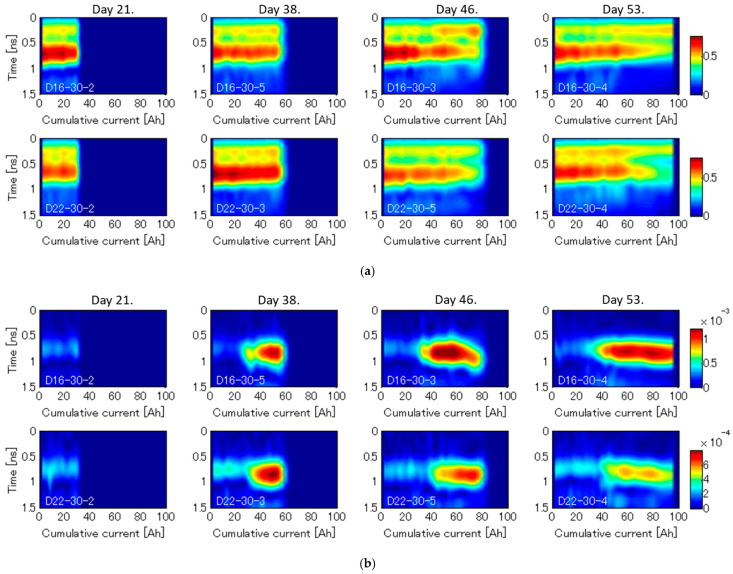
Monitoring results obtained by VDR while the electrolytic corrosion test in the case of D16 rebar for: (**a**) non-modulation components; (**b**) Doppler modulation components. The enveloped waveforms are aligned in the lateral direction as a function of the cumulative current with the amplitude representing in color scale. The amplitude of the Doppler component is multiplied by 1000.

This section discusses the monitoring results of VDR measurement for each test piece while the electrolytic corrosion test. Radar waveforms were obtained by taking inverse Fourier transformation after applying a Gaussian band-pass filter with a center frequency of 2 GHz and a bandwidth of 2 GHz. Figure 13a,b show the radar profile for non-modulation and Doppler modulation component, respectively. The enveloped waveforms are aligned in the lateral direction as a function of the cumulative current with the amplitude representing in color scale. In the non-modulation component, the reflected wave around 0.75 ns corresponds to the reflection from the rebar. The amplitude of the reflected wave does not significantly change and slightly decreases with the cumulative current increasing. This information can even be obtained by a conventional RC radar. On the other hand, the Doppler component abruptly and significantly increases in more than 30 Ah. Since corrosion cracks are seemed to occur at around 30 Ah as shown in Figure 12, it corresponds to the timing of increment of Doppler components. This information can be obtained by only VDR and can never be obtained by a conventional RC radar.

**Figure 14:**
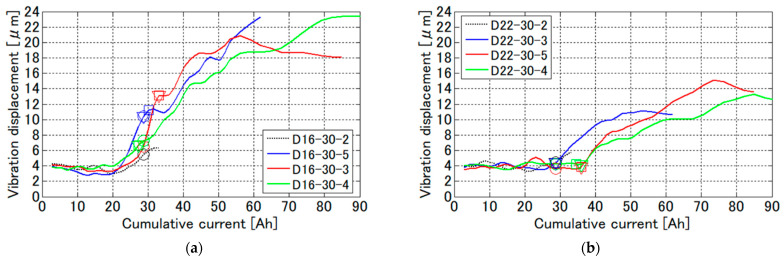
Rebar vibration displacement measured by VDR in the electrolytic corrosion test for: (**a**) D16 rebar; and (**b**) D22 rebar. The markers indicated by a circle, rectangle, and triangle mean the timing at occurrence of cracks on the top surface, that on the side surfaces of the concrete, and leakage of rust water from the crack, respectively.

Figure 14 shows the results of the dependence of the rebar vibration displacement on the cumulative current. The circle, squares and triangle marks in the figure indicate the timing when occurrence of cracks on the top surface, that on the side surfaces, and rust leakage from the cracks were observed, respectively. When the cumulative current is less than 20 Ah, the vibration displacement has almost constant value of around 4 μm. After that, the vibration displacements abruptly start to increase around 20 Ah and 30 Ah in the case of D16 and D22 rebar, respectively. The abrupt increase of the vibration displacement is highly reproducible. The increase rate in D22 rebar is smaller than that in D16 rebar because thicker the rebar diameter is, smaller the production corrosion per unit area is. The cumulative current when the vibration displacement starts to increase is smaller than that when the corrosion cracks start to appear on the concrete surface, suggesting that this method has possible to detect the indication of rebar corrosion in advance of occurrence of corrosion cracks.

## 5. Discussion

The vibration displacement of the rebar embedded in a concrete is successfully obtained as a function of the cumulative current while the electrolytic corrosion test. The corrosion loss of the rebar is simultaneously estimated also the function of the cumulative current with Equations (14) and (15). The corrosion amount per a unit cross-sectional area can be calculated with the rebar diameter and the effective length of the corroded area.

**Figure 15:**
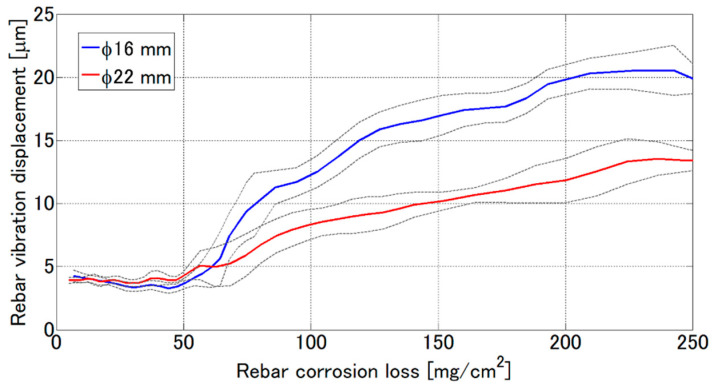
Relationship between rebar corrosion loss and rebar vibration displacement obtained by VDR. The grey dotted lines mean standard deviation.

Figure 15 shows the relationship between the corrosion amount per a unit cross-sectional area and the vibration displacement obtained by the VDR. The solid and broken line corresponds to for the D16 and D22 rebar, respectively. The grey solid line represents the standard deviation of the available vibration displacements in five test pieces. The vibration displacement is almost same up to 50 mg/cm^2^, so it is difficult to evaluate the corrosion amount from the vibration displacement in the corrosion range of less than 50 mg/cm^2^. On the other hand, the estimated corrosion loss monotonically increases in the corrosion range of more than 50 mg/cm^2^ with the increasing vibration displacement. The vibration displacement at the corrosion loss of 250 mg/cm^2^ reaches to 4 and 2.5 times higher than that of the healthy rebar of D16 and D22, respectively.

It is known that the corrosion in natural situations is different from that in an electrolytic corrosion tests. In addition, rebars in real world concrete structures experience stresses that could be not far from their yielding stress while in the performed tests no tension was applied, so the Equations (14) and (15) are applicable in the laboratory tests and cannot always be applied to practical situations. Therefore, it seems to be currently difficult to quantitatively estimate the rebar corrosion loss in an actual situation by measuring the rebar vibration displacement with VDR. These are problems to be resolved for application of VDR to practical situations in future.

On the other hand, this method has a great advantage of independence from the moisture in concrete because the vibration displacement can be obtained by an amplitude ratio of the two kinds of radar reflection response in which the electromagnetic wave propagates through the same path. Moreover, this method also has an advantage that the rebar vibration displacement has higher contrast between corroded and healthy rebar than other evaluation methods, so it seems to be useful to employ the rebar vibration displacement as a threshold level of the corrosion stage of rebar in practical cases. The vibration displacement will depend on the rebar diameter, the cover thickness of the rebar, strength of the magnetic field and so on. If their dependency can be modeled and calculated in actual cases, we can determine the standard value of the vibration displacement for healthy rebars in various practical situations. If we cannot judge the corrosion stages by the absolute threshold level, the spatial variation of the vibration displacement can be easily measured by scanning VDR along a rebar. This makes it possible to judge the corrosion situation relatively by comparing results measured in a non-corroded area in situ.

Finally, disadvantages of this method are discussed. The first one is that this method needs a high dynamic range because Doppler components are around −60 dB smaller than an unmodulated component in the case that the rebar with the concrete cover of 40 mm has the vibration displacement of 5 μm, so much more vibration force would also be needed for deeper rebar. The second one is measurement speed. The IF bandwidth of the network analyzer should be set to be much less than the vibration frequency. Since it is set to be 10 Hz in this system, it took 120 s for one measurement. However, it is expected that these problems can be solved by introducing a pulse radar system to the VDR measurement system.

## 6. Conclusions

This paper proposed a vibro-Doppler radar (VDR) as a new nondestructive evaluation (NDE) method of rebar corrosion loss in concrete. The developed VDR system can forcibly vibrate the rebar in concrete with an excitation coil and quantitatively measure the vibration displacement of the rebar in concrete. The validity of the vibration unit of this system was demonstrated by a laser displacement sensor. The rebar vibration displacement was able to successfully be measured in concrete. It is clear that the moisture of concrete does not significantly affected the rebar vibration displacement in the VDR measurement. The monitoring of the rebar vibration displacement was performed during an electrolytic corrosion test. The relationship between the rebar corrosion loss and the rebar vibration displacement obtained by VDR was examined. As the rebar corrosion increases, the vibration displacement increases and reached a value four times higher than that in the healthy condition at the corrosion loss of 250 mg/cm^2^.

Compared to other corrosion evaluation methods including conventional GPR, this method is less affected to the moisture content in the rebar corrosion evaluation and has the possibility of early detection of corrosion and a more quantitative evaluation of corrosion. On the other hand, the disadvantages are the necessity of a high dynamic range radar system and time-consuming measurements.

## Figures and Tables

**Table 1:** Vibration displacement measured in air by LDS.

Excitation Current [A]	Vibration Displacement [μm]
8	6.7
9	8.6
10	11.5

**Table 2:** Measurement setting of network analyzer and characteristics of image canceling mixer.

Instrument	Item	Value
Vector network analyzerRohde & Schwarz ZVL-13	Center frequency	5 GHz
	Span	8 GHz
	Power	−8 dBm
	IF bandwidth	10 Hz
	Number of points	151
Image canceling mixer	Output power	25 dBm
	Bandwidth	1.5–12 GHz

**Table 3:** Concrete mixing and fresh property of the concrete, and averaged mechanical property of the concrete test piece wet-cured for 28 days.

Water	Cement	W/C	Coarse Aggregate	Fine Aggregate	Fine Aggregate Ratio	Air	Slump	Dry Density	Compressive Strength
[kg/m^3^]	[kg/m^3^]	[%]	[kg/m^3^]	[kg/m^3^]	[%]	[%]	[cm]	[Kg/m^3^]	[N/mm^2^]
168	305	55	947	864	48	4.5	12	2329	41.2

**Table 4:** Corrosion loss of the rebar extracted after the electrolytic corrosion test.

Test Piece No.	Cover of Concrete [mm]	Diameter of Rebar [mm]	Period of Electrolytic Corrosion Test [Day]	Cumulative Current [Ah]	Amount of Corrosion Loss [g]	Corrosion Loss [%]
D16-30-1	30	16	14	20	6.2	2.1
D16-30-2			21	30	8.1	2.7
D16-30-5			38	55	19.3	6.5
D16-30-3			46	77	32.9	11.1
D16-30-4			53	98	43.0	14.6
D22-30-1		22	14	20	4.3	0.8
D22-30-2			21	30	7.2	1.3
D22-30-3			38	55	17.6	3.0
D22-30-5			46	77	33.8	6.0
D22-30-4			53	98	44.3	7.7

## Data Availability

The data presented in this study are available on request from the corresponding author.
